# Diagnostic accuracy of the 1,3-beta-d-glucan test and lactate dehydrogenase for pneumocystis pneumonia in non-HIV patients

**DOI:** 10.1038/s41598-021-88729-z

**Published:** 2021-04-29

**Authors:** Ruixue Sun, Dan Lv, Meng Xiao, Li Zhang, Jun Xu, Xuezhong Yu, Huadong Zhu, Jing Yang

**Affiliations:** 1 Emergency Department, State Key Laboratory of Complex Severe and Rare Diseases, Peking Union Medical College Hospital, Chinese Academy of Medical Science and Peking Union Medical College, Beijing, China; 2 Beijing Emergency Medical Centre, Beijing, China; 3 Laboratory Department, State Key Laboratory of Complex Severe and Rare Diseases, Peking Union Medical College Hospital, Chinese Academy of Medical Science and Peking Union Medical College, Beijing, China

**Keywords:** Microbiology, Infectious-disease diagnostics

## Abstract

We evaluated the serum levels of (1–3)-beta-d-glucan (BG) and lactate dehydrogenase (LDH) as a tool to support pneumocystis pneumonia (PCP) diagnostic procedures in non-HIV patients. We retrospectively collected non-HIV (human immunodeficiency virus) patients presenting clinical features of PCP between April 1st, 2013, and December 31st, 2018. A total of 225 included patients were tested for *Pneumocystis jirovecii* by polymerase chain reaction (PCR) and methenamine silver staining. Based on different exclusion criteria, 179 cases were included in the BG group, and 196 cases were included in the LDH group. In each group, cases with positive immunofluorescence (IF) microscopy and PCR were considered proven PCP, while cases with only positive PCR were considered probable PCP. Fifty patients with negative IF and PCR results and proven to be non-PCP infection were chosen randomly as the control group. The cut-off levels of BG and LDH to distinguish non-PCP from probable PCP were 110 pg/mL and 296 U/L with 88% sensitivity and 86% specificity, and 66% sensitivity and 88% specificity, respectively. The cut-off levels of BG and LDH to distinguish non-PCP from proven PCP were 285.8 pg/mL and 379 U/L with 92% sensitivity and 96% specificity, and 85% sensitivity and 77% specificity, respectively. The cut-off levels of BG and LDH to distinguish non-PCP from proven/probable PCP were 144.1 pg/mL and 363 U/L with 90% sensitivity, 86% specificity and 80% sensitivity, 76% specificity respectively. BG and LDH are reliable indicators for detecting *P. jirovecii* infection in HIV-uninfected immunocompromised patients.

## Introduction

Pneumocystis pneumonia (PCP) is a potentially life-threatening infection that occurs in immunocompromised patients. HIV-infected patients are at the highest risk of PCP. In our hospital, nearly half of the population who at high risk of PCP infection are patients with rheumatic immune diseases, because they have to receive long-term treatment with glucocorticoids and other immunosuppressive drugs. As reported, other non-HIV immunocompromised patients include haematopoietic stem cell recipients, solid organ transplant recipients and patients with cancer^1^. As PCP causes serious morbidity, timely diagnosis means better prognosis for these patients^2^.

Non-HIV immunosuppressed patients with PCP typically present with hypoxaemia associated with fever, dry cough and significant dyspnoea^1,3^. The radiographic findings in PCP are nonspecific; in some patients, HRCT may reveal an extensive ‘ground-glass’ appearance or cystic lesions. Although clinical and radiographic findings can be highly suggestive of a diagnosis of PCP in immunocompromised patients, the definitive diagnosis of PCP requires identification of the organism either by tinctorial staining, fluorescent antibody staining, or polymerase chain reaction (PCR)-based assays of respiratory specimens^3^. However, as patients normally have a dry cough, respiratory specimens should be collected in specific infectious disease clinics or obtained by bronchoalveolar lavage. These two methods are difficult to implement. Thus, the 1,3-beta-d-glucan (BG) test and lactate dehydrogenase (LDH), which are more readily available by blood tests, may be used to help support the diagnosis of PCP^3,4^.

Beta-d-glucan is the major component of *P. jirovecii’s* cyst wall^5^. Several studies have demonstrated that a high level of BG is a discriminative marker of PCP in immunocompromised patients, but the target population is HIV-infected patients^5–9^ or HIV-infected patients mixed with non-HIV-infected patients^10–13^. Only a few studies have merely focused on non-HIV-infected patients, and the main underlying conditions of these patients are malignancy or solid organ transplantation^8,14^^.^ However, prospective data about the use of BG for diagnosing PCP in non-HIV-uninfected patients are limited. As HIV-related PCP and non-HIV-related PCP have different clinical characteristics and prognoses^15,16^, whether the observations related to the accuracy of BG as a diagnostic marker for PCP in HIV-infected individuals can be extrapolated to the non-HIV population is still a question. In addition, LDH is regarded as a non-specific marker of PCP because it is possibly caused by the underlying lung inflammatory responses and damage^3^. In Deng C’s study, the sensitivity of LDH in the diagnosis of PCP was above 80% in the HIV group^17^. We are interested in its sensitivity and specificity in the HIV-negative group. In our study, we explored the ability of serum BG and LDH to support the diagnosis of PCP in non-HIV conditions.

## Methods

### Study design

**Figure 1:**
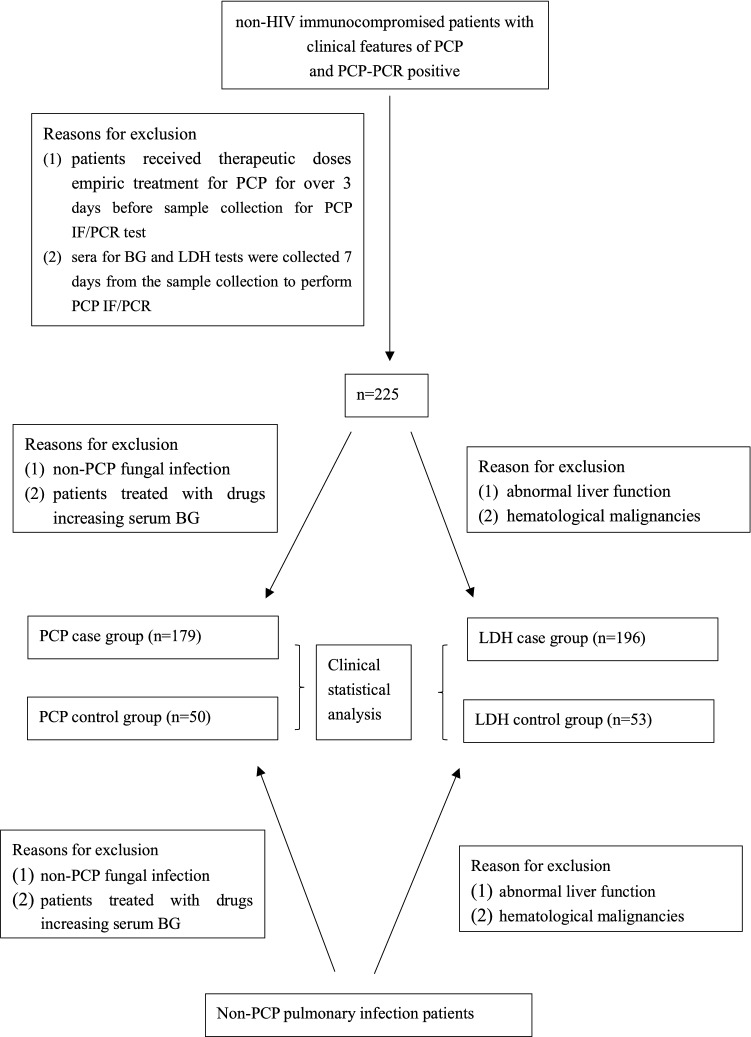
patients sample flow. *HIV* human immunodeficiency virus, *PCP* pneumocystis pneumonia, *PCR* polymerase chain reaction, *IF* immunofluorescence, *BG* (1–3)-beta-d-glucan, *LDH* lactate dehydrogenase.

Peking Union Medical College Hospital receives hundreds of non-HIV immunocompromised patients from mainland China. We conducted a retrospective study in our hospital. All patients admitted with a diagnosis of PCP from April 1st, 2013, to December 31st, 2018, were screened for eligibility. The inclusion criteria were as follows: (1) presenting one or more of the following clinical features: fever, dry cough, hypoxaemia, and CT scan demonstrating bilateral infiltrates or ground glass; (2) respiratory secretions were tested by methenamine silver staining and polymerase chain reaction (PCR) to detect *P. jirovecii*; and (3) HIV tests were negative. The exclusion criteria were as follows: (1) patients received therapeutic doses of empiric treatment for PCP for over 3 days before sample collection to perform PCP IF/PCR tests; (2) sera for BG and LDH tests were collected 7 days from sample collection to perform PCP IF/PCR; (3) patients with non-PCP fungal infection or those undergoing therapies may have increased serum BG levels (such as albumin, intravenous immunoglobin, haemodialysis, etc.) and cannot be included in the BG group; and (4) abnormal liver function or haematological malignancies were not included in the LDH group. In each case group, cases with methenamine silver stain and PCR were considered proven PCP; if only the PCR was positive, they were considered probable PCP. Patients with negative methenamine silver staining and PCR results that proved to be non-PCP infections were chosen randomly as the control group, and they also matched the exclusion criteria of the BG case group and LDH case group (Fig. 1: patient sample flow).

From patient records, we collected patient information, including age, sex, length of hospital stays, ICU stay, risk factors (autoimmune disease, haematological malignancies, solid tumour, organ transplantation, bone marrow transplantation), therapy history (glucocorticoid, immunosuppressant and preventive dose of sulfanilamide), clinical manifestation (fever, cough, wheeze), chest CT features, level of BG and LDH, methenamine silver stain and PCP PCR test results, and prognosis. Finally, the level of BG and the LDH value on PCP diagnosis were analysed, and the clinical features of the cases were summarized at the same time.

### Laboratory analyses

Serum BG levels were determined by kinetic chromogenic kit (fungal (1–3)-β-d glucan detection kit produced by CHARLES RIVER Company, Zhanjiang, China), and the test was performed in duplicate where the normal range of BG is < 100.5 pg/mL. LDH levels were determined using the quantitative Lactate Dehydrogenase Assay Kit (BECKMAN, USA), where the normal range is 0–250 U/L. The pneumocystis is detected by immunofluorescence microscopy after Grocott methenamine silver stain(GMS). The process of GMS is described below, and IF (the kit was Gold Star Taq Man Mixture provided by CoWin Biosciences) microscopy was performed directly after the GMS. *P. jirovecii* DNA was detected in respiratory secretions by an in-house quantitative real-time PCR. BG and LDH testing was performed in a blinded manner to the IF microscopy and PCR results.

#### Grocott methenamine stain

Formalin-fixed samples on glass slides were incubated with periodic acid for 10 min, then stained by silver solution for 1.5 h in a water bath at 56℃, and stained with 0.25% gold chloride solution, 3% sodium thiosulfate, brilliant green by turn for 1 min respectively, then the result was read. The slides were rinsed with water after every step^18^.

#### Pneumocystis jirovecii DNA detection

*Pneumocystis jirovecii* DNA was detected by real-time PCR. The respiratory sample was mixed with 4% NaOH solution, and 1 mL of the liquefaction was placed in an experimental tube and centrifuged at 12,000 rpm/min for 5 min. Then, the supernatant was discarded. The remaining liquefaction was mixed with normal saline and centrifuged again at the same speed for 1 min, and then the supernatant was discarded. Next, 50 μL of magnetic beads (Da An Gene Co., Ltd. of Sun Yat-sen University) was added to the residue and incubated for 5 min at 100 °C after oscillating centrifugation, and used for real-time PCR amplification immediately. *P. jirovecii*-specific primer and fluorescent probe were designed targeting mitochondrial larger subunit (mtLSU) rRNA region, and human albumin gene was used for internal control^18^.

The interpretation of the results is as follows: (1) positive: the fluorescence signal increased significantly, and the amplification curve was S-shaped, with a Ct value ≤ 37; (2) negative: the fluorescence signal did not increase and showed an S-shape; (3) grey area: the fluorescence signal increased significantly, and the amplification curve was S-shaped, with a Ct value > 37. The grey area result needs to be repeated; if the confirmatory result is positive or still in the grey area, the final result is weakly positive, and if the confirmatory result is negative, the final result is negative. In the study, both positive and weekly positive results were treated as “PCP-PCR positive”, and a negative result was treated as “PCP-PCR negative”^18^^.^

### Statistics

Numerical variables are provided as the mean (standard deviation [SD]) and median (range, interquartile range [IQR]), and categorical data are described by absolute numbers and frequencies. Numerical variables were analysed by the unpaired t-test or Mann–Whitney test for two groups or by one-way ANOVA with post hoc pairwise comparisons by LSD’s test for more than two groups. Categorical variables were compared by χ^2^ or Fisher’s exact test. To evaluate test performance, sensitivity and specificity were calculated with 95% confidence intervals. The positive likelihood ratio (P-LR) was calculated as sensitivity/(1 − specificity), and the negative likelihood ratio (N-LR) was calculated as (1 − sensitivity)/specificity with 95% confidence intervals. The percentage agreement between the test results and Cohen’s kappa coefficient (K) was also calculated. For correlation analysis, a scatterplot was constructed, and Spearman’s rank correlation coefficient (rs) was calculated^12^. Statistics Analysis Software version 9.4 (SAS INSTITUTE INC, Cary, NC, USA) was used at a significance level of 0.05 in Figs. 4 and 5.

### Data description and repository

Patient information was collected through the HIS system of Peking Union Medical College Hospital (accession number 11578).

### Ethics approval and consent to participate

The Institutional Review Board (IRB) of Peking Union Medical College Hospital reviewed the study and determined that it is a retrospective study and that the design is scientifically sound and meets the ethics standards. The IRB thus approved the study.

### Consent for publication

Consent for publication has been obtained from the patients reported in this article.

### Statement

All methods in the study were carried out in accordance with relevant guidelines and regulations.

### Consent for participate

Informed Consent has been obtained from the patients reported in this article. For patients < 18 years old, informed Consent was obtained from parents and/or legal guardian.

## Result

The BG group and LDH group included 179 and 196 patients, respectively, and descriptive characteristics are found in Tables 1 and 2. These results showed that the average age of both proven and probable PCP patients was 50 years old, and the male to female ratio was almost 1:1. In addition to systemic lupus erythematosus (SLE) and other rheumatic immune diseases, interstitial lung diseases and glomerulonephritis without definitive cause were common underlying conditions. The percentages of proven and probable PCP patients admitted to ICU were 49.7% and 46.4% in BG group and in LDH group respectively. In BG group, the mortality rates of proven PCP patients and probable PCP patients were 25.0% and 18.1%, and in LDH group, the mortality rates of proven PCP patients and probable PCP patients were 24.2% and 15.3%.

**Table 1:** Patient characters of BG groups.

	Proven PCP (n = 24)	Probable PCP (n = 155)	Negative PCP (n = 50)
Male/female ratio	1:1	1.21:1	1.53:1
Age (years), media (range)	50 (21–83)	50 (15–83)	59(18–84)
**Biological samples type**			
Bronchoalveolar lavage fluids (n, %)	10 (41.67)	40 (25.81)	21 (42.00)
Induced sputa (n, %)	14 (58.33)	115 (74.19)	29 (58.00)
**Underlying condition**			
SLE (n, %)	5 (20.83)	18 (11.61)	–
non-SLE RID (n, %)	10 (41.67)	54 (34.84)	–
Hematological malignancy (n, %)	2 (8.33)	8 (5.16)	–
Others (n, %)	7 (29.17)	75 (48.39)	–
**Outcome of PCP**			
LOH (days), media (range)	25 (3–80)	24 (2–101)	–
ICU (n, %)	11 (45.83)	78 (50.32)	–
30-day mortality, (n, %)	6 (25.00)	28 (18.10)	–

*SLE* systemic lupus erythematosus, *RIG* rheumatic immunity diseases, *LOH* length of hospitalization, *ICU* intensive care unit.

**Table 2:** Patient characters of LDH groups.

	Proven PCP (n = 33)	Probable PCP (n = 163)	Negative PCP (n = 53)
Male/female ratio	1.06:1	0.94:1	1.44:1
Age (years), media (range)	50 (21–72)	50 (18–83)	58 (18–84)
**Biological samples type**			
Bronchoalveolar lavage fluids (n, %)	10 (30.30)	43 (26.38)	23 (39.66)
Induced sputa (n, %)	23 (69.70)	120 (73.62)	30 (60.34)
**Underlying condition**			
SLE (n, %)	5 (15.15)	18 (11.04)	–
non-SLE RID (n, %)	11(33.33)	62 (38.04)	
Others (n, %)	17 (51.52)	82 (50.31)	–
**Outcome of PCP**			
LOH (days), media (range)	33 (3–79)	20(2–101)	–
ICU (n, %)	14 (42.42)	77 (42.24)	–
30-day mortality, (n, %)	8 (24.24)	25 (15.34)	–

**Figure 2:**
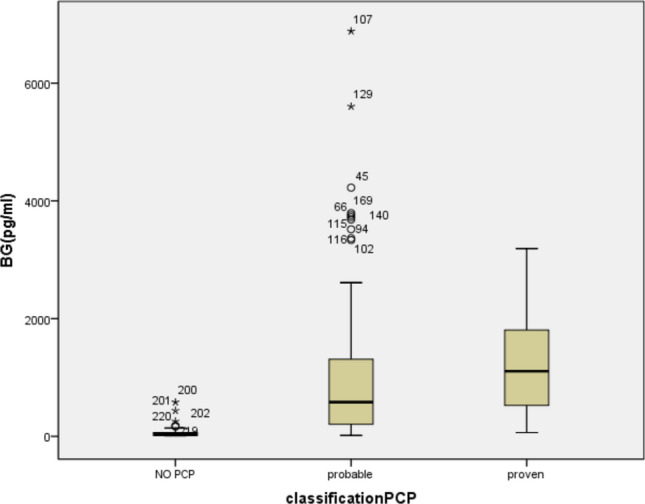
(Box-and-whisker plot): The mean levels of BG in patients with no PCP, probable PCP and proven PCP were 65.03 pg/mL, 983.22 pg/mL, and 1220.09 pg/mL, respectively. The medians of the 3 groups were 27.95 (14.60, 65.00) pg/mL, 580.0(206.0, 1311.0) pg/mL,1107.0 (524.1, 1806.0) pg/mL respectively.

**Figure 3:**
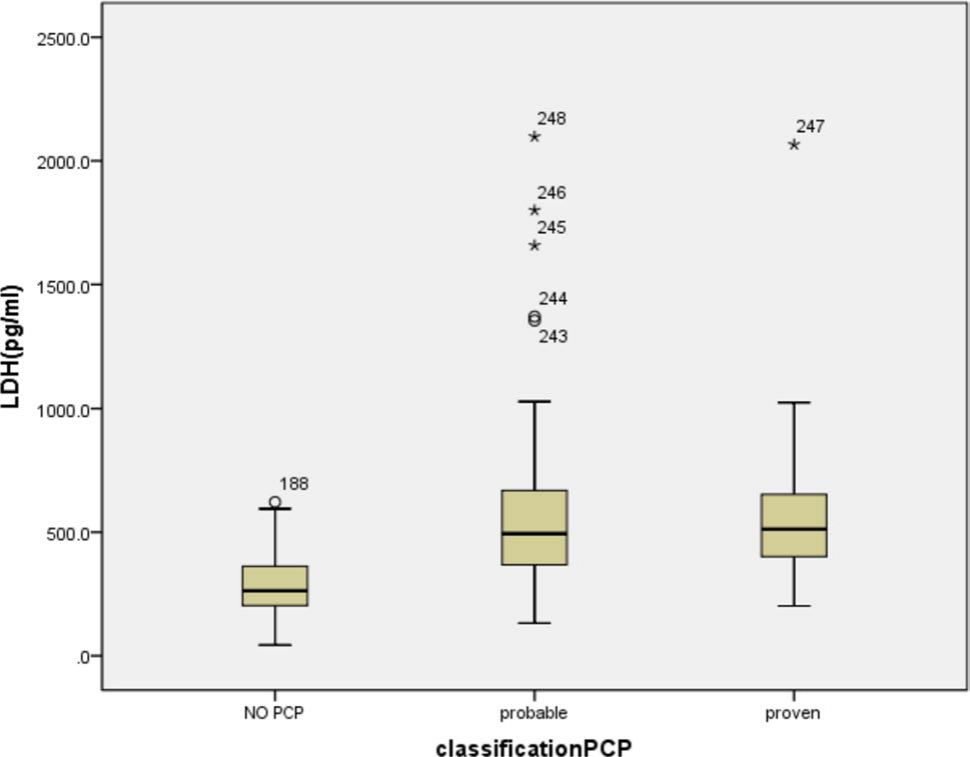
(Box-and-whisker plot): The mean levels of LDH in the no PCP, probable PCP and proven PCP groups were 297.62 U/L, 547.88 U/L, and 854.23 U/L. The medians of the 3 groups were 263.0 (203.0, 302.0) pg/mL, 494.0 (367.0, 672.0) pg/mL, 512.0 (401.0, 653.0) pg/mL respectively.

The mean levels of BG in patients with no PCP, probable PCP and proven PCP were 65.03 pg/mL, 983.22 pg/mL, and 1220.09 pg/mL, respectively (Supplementary Table 1 and Fig. 2: The mean levels of BG in the no PCP, probable PCP and proven PCP groups), and the mean levels of LDH in the three groups were 297.62 U/L, 547.88 U/L, and 854.23 U/L (Supplementary Table 2 and Fig. 3: The mean levels of BG in the no PCP, probable PCP and proven PCP groups). Except for the BG level between the probable PCP group and proven PCP group, the levels of BG and LDH between each pair of groups were significantly different (Supplementary Tables 3, 4).

**Figure 4:**
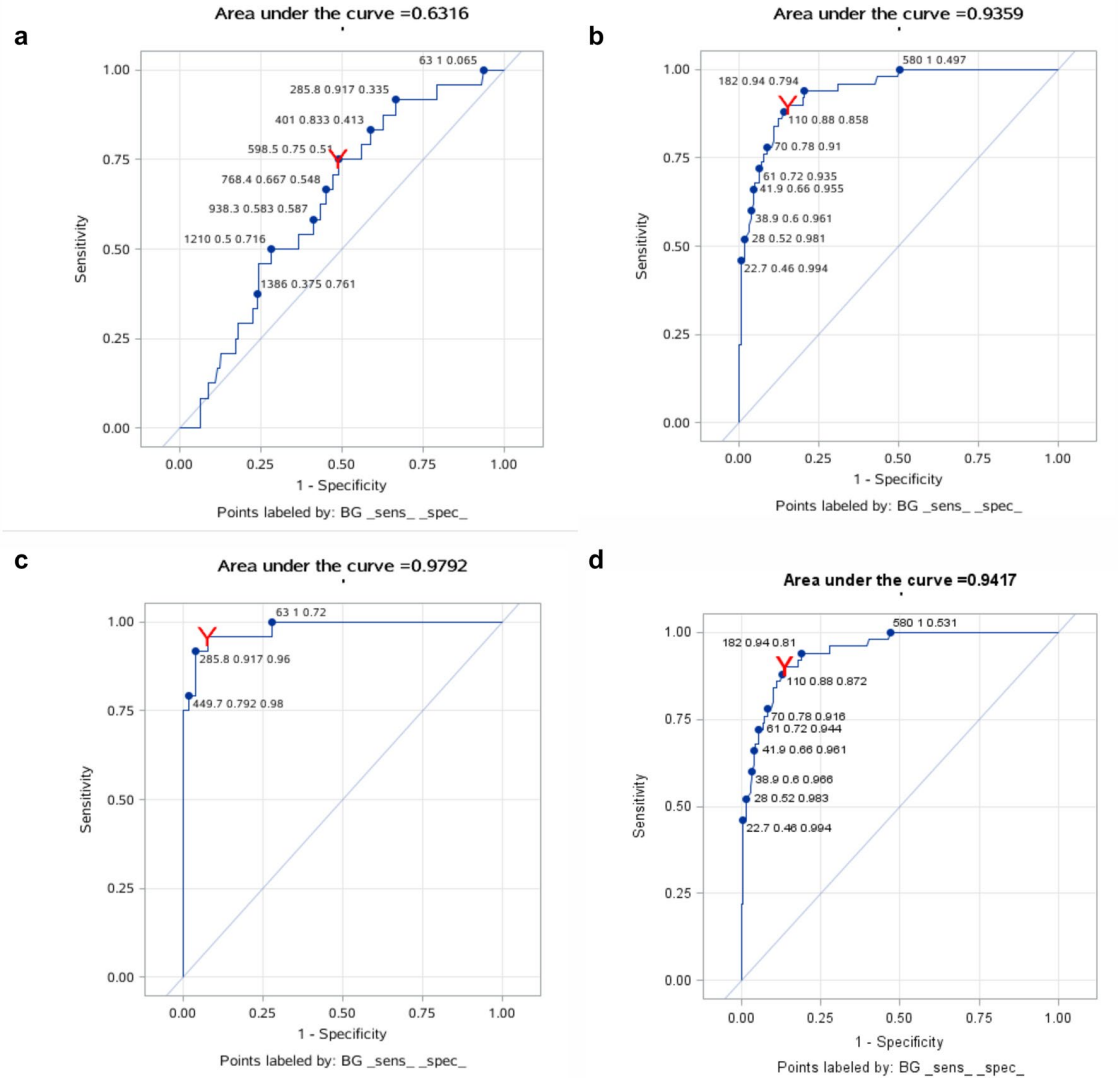
(**a**) The ROC curves for BG of probable PCP and proven PCP groups; (**b**) The ROC curves for BG of probable PCP and NO PCP groups; (**c**) The ROC curves for BG of proven PCP and NO PCP groups; (**d**) The ROC curves for BG of proven/probable PCP and NO PCP groups.

**Figure 5:**
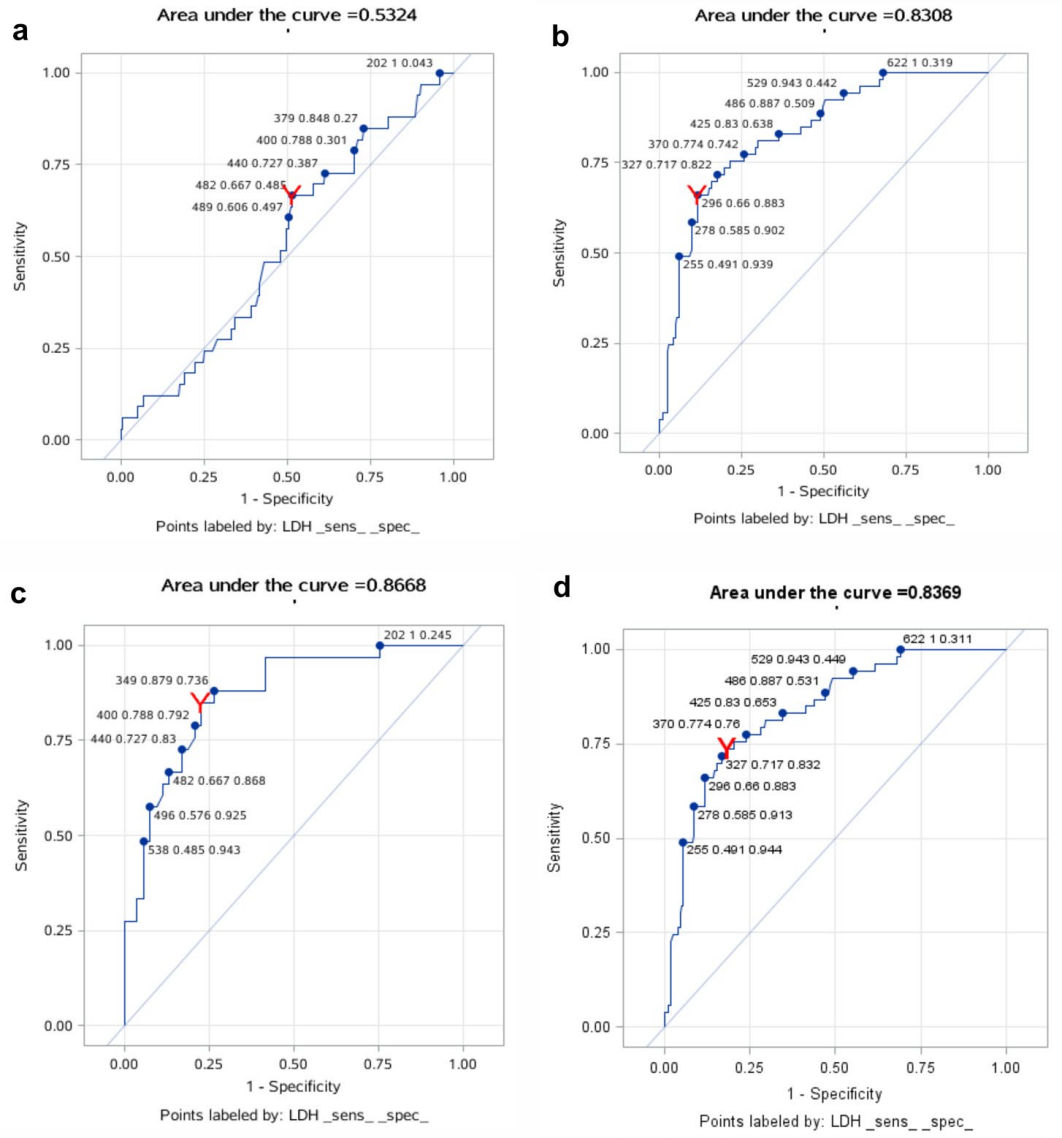
(**a**) The ROC curves for LDH of probable PCP and proven PCP groups; (**b**) The ROC curves for LDH of probable PCP and NO PCP groups; (**c**) The ROC curves for LDH of proven PCP and NO PCP groups; (**d**) The ROC curves for LDH of proven/probable PCP and NO PCP groups.

The ROC curves for BG and LDH are shown in Figs. 4a–d and 5a–d. From the analysis of Supplementary Tables 5 and 6, we can see the diagnostic value of BG and LDH for PCP diagnosis: the cut-off levels of BG and LDH to distinguish no PCP from probable PCP were 110 pg/mL and 296 U/L with 88% sensitivity and 86% specificity, and 66% sensitivity and 88% specificity, respectively. The cut-off levels of BG and LDH to distinguish no PCP from proven PCP were 285.8 pg/mL and 379 U/L with 92% sensitivity and 96% specificity, and 85% sensitivity and 77% specificity, respectively. The cut-off levels of BG and LDH to distinguish no PCP from proven/probable PCP were 144.1 pg/mL and 363 U/L with 90% sensitivity and 86% specificity, and 80% sensitivity and 76% specificity, respectively. However, neither BG nor LDH was good at distinguishing between probable PCP and proven PCP.

## Discussion

To our knowledge, the definitive diagnosis of PCP is still challenging. Most importantly, the organism cannot be cultured in clinical laboratories, so the diagnosis depends on examination of respiratory secretions usually obtained by invasive bronchoscopy, and visualization of the organism in respiratory secretions requires special staining techniques^19^. Beta-glucan and lactate dehydrogenase, as non-invasive markers, can provide useful, non-invasive adjunctive diagnostic tools. In this retrospective observational study, the usefulness of serum BG and LDH in the diagnosis of PCP in non-HIV patients was assessed. The results supported that these two biomarkers have excellent power for discriminating proven PCP or probable PCP from non-PCP patients. Serum BG and LDH tests may be incorporated in the diagnosis of PCP in non-HIV immunocompromised patients with fever, dyspnoea and infiltrates on chest CT scans.

Several previous publications have studied BG as a diagnostic tool for PCP in immunocompromised patients. We reviewed the recent literature on serum BG as a marker for PCP (Table 3)^3,5–7,9–11,13,20–29^, and there have been few studies on non-HIV patients. Mark et al. summarized studies before 2009 and found that elevated serum BG was a reliable indicator for the diagnosis of PCP in solid organ transplantation and haematological malignancy patients^8^. In 2019, Sejal evaluated 53 cases of almost the same population and came to the same conclusion^14^. Some authors mentioned that the burden of *P. jirovecii* organisms in the lungs of HIV PCP patients is usually heavier than that in non-HIV PCP patients^30^; thus, we studied the reliability of the BG test for the diagnosis of PCP in non-HIV immunocompromised patients. In our study, patients’ underlying conditions mainly included rheumatic immune diseases, interstitial lung diseases and glomerulonephritis without a definitive cause, and BG was also a reliable indicator for PCP.

**Table 3:** An overview of recent literatures on serum BG as a marker for PCP.

1st Author	Year	Journal	N (PCP+) HIV+/HIV−	Main result
Maria^5^	2009	Clin Vaccine Immunol	23/8	The medium BG value of PCP and NPCP group were 423 pg/mL and 60 pg/mL respectively
Esteves ^6^	2014	Eur J Clin Microbiol Infect Dis	100/0	The most promising cut-off levels for diagnosis of PCP were determined to be 400 pg/mL of BG and 350 U/L of LDH, which combined with clinical data presented 92.8% sensitivity, 83.9% specificity
Helena ^7^	2019	BMC Infect Dis	21/0	Beta glucan with cut-off level 200 pg/mL combined with a positive Pneumocystis jirovecii PCR result had sensitivity and specificity of 92 and 90%, respectively in PCP diagnosis
Mark^8^	2011	J Infection	0/21	Elevated serum b-d-glucan was a reliable indicator for PCP with a sensitivity of 0.90 and specificity of 0.89 at the 60 pg/mL cut-off
Salerno ^9^	2014	Respir Med	119/0	BG yielded a sensitivity of 91% and specificity of 92% for PCP diagnosis at a 300 pg/mL cut-off level
Engsbro^12^	2019	Med Mycol	NA	The Sensitivity and specificity were 87% and 70% when the level of BG was 95 pg/mL
Esteves^13^	2015	Clin Microbiol Infect	89/34	BG was found to be the most reliable serologic biomarker for PCP diagnosis with sensitivity 91.1%, Specificity 71.6%, followed by KL-6, LDH and SAM. The BG/KL-6 combination test was the most accurate serologic approach for PCP diagnosis, with 94.3% sensitivity and 89.6% specificity
Sejal^14^	2019	Clin infect dis	0/53	With PCP PCR alone as the reference method, BG (≥ 80 pg/mL) had a sensitivity of 69.8% and a specificity of 81.2% for PCP. At ≥ 200 pg/mL, in patients with a positive PCR and a compatible PCP clinical syndrome, BDG had a sensitivity and a specificity of 70% and 100%
Akira^21^	2011	J Clin Microbiol	–	This meta-analysis of twelve studies for PCP from January 1966 to November 2010 showed BG had had a sensitivity of 96% and a specificity of 84% for PCP
Brian R^22^	2013	AIDS	129/0	The sensitivity of BG for PCP in HIV-participants with respiratory symptoms was 92.8% (95% CI 87.2–96.5%), and specificity 75.0% (50.9–91.3%) at the cutoff of 80 pg/mL
Karageorgopoulos^23^	2013	Clin Microbiol Infect	–	Fourteen studies were included in the meta-analysis. BG data were analysed for 357 PCP cases and 1723 controls. The average sensitivity and specificity of BG were 94.8%and 86.3%, respectively
Hyo-Ju^24^	2017	Plos ONE	0/50	The mean ± SD of the concentration of BG in the patients with PCP were similar to those of patients with candidemia and chronic disseminated candidiasis, but higher than those of patients with invasive aspergillosis, mucormycosis and tuberculosis as well as healthy volunteers
J. Held^25^	2011	Clin Microbiol Infect	17/33	Serum BG is an excellent diagnostic performance with a sensitivity of 98.0% and a specificity of 94%. BG did not correlate with the outcome of patients or with the *P. jirovecii* burden
Karl^26^	2018	J Clin Microbiol	36/74	BG identified 66/73 patients with positive qPCR tests for an overall sensitivity of 91%, and it was negative in 25/25 controls with negative qPCR for a specificity of 100% using the predefined cut-off 11-pg/mL
Paul^27^	2011	Clin Infect Dis	173/0	The sensitivity of b-glucan dichotomized at 80 pg/mL for the diagnosis of PCP was 92% (95% confidence interval [CI] 87–96%), and the specificity was 65%
Tamayo^28^	2009	Clin Infect Dis	111/0	A b-d-glucan cut-off value of 23.2 pg/mL had a sensitivity of 96.4% and a specificity of 87.8% in PCP diagnosis
Wei-Jie^29^	2015	J Thorac Dis	–	The meta-analysis of 13 studies estimates for serum-BG assay for definite PCP were as follows: Se 0.91 [95% confidence interval (CI) 0.88–0.93]; Sp 0.75 (95% CI 0.68–0.81)

In studies of HIV-positive patients, the cut-off levels of BG were 80 pg/mL, 200 pg/mL, 300 pg/ml and 400 pg/mL^6,7,9,21^. The cut-off values in non-HIV patient studies were also volatile^8,20,24^; in this study, the cut-off value of BG was 110 pg/mL. As mentioned before, we suspected that the difference was due to the different numbers of *P. jirovecii* organisms in different patients. However, J. Held proved that BG did not correlate with the *P. jirovecii* burden^25^. More data and research are required for a precise range of BG levels to diagnose PCP.

There were 23 false-positive cases in this study, and the reasons are chiefly as follows: the main reason is that beta-glucan is not a species-specific marker for *P. jirovecii*, so the BG test in the care of immunocompromised pneumonia is “pan-fungal” in nature. Some invasive fungal infections, including candidaemia, aspergillosis, fusariosis, trichosporiosis, and histoplasmosis, may trigger a high level of BG^27^. Although we excluded patients with certain non-PCP fungal infections, on review, 9 patients were treated with nystatin for oral candidiasis, and 4 patients received voriconazole as an empirical therapy without clear evidence of fungal infection. Immunocompromised patients are at high risk of fungal infection, so occult fungal infection may affect the results. Although several studies have shown that the serum BG levels of patients with invasive fungal infections are generally lower than those with PCP^13,24^, when these two types of opportunistic infections are mixed, they are difficult to identify. In addition, false-positive BG results can occur in patients on haemodialysis, with gram-negative bacteraemia, severe mucositis, or with the use of some antibiotics and intravenous immunoglobulins^3,31^. In our cases, the only factor was the use of antimicrobials, which mainly included third-generation cephalosporins, carbapenems and cefoperazone sulbactam; however, the effects of these antibiotics on serum BG are still unclear. On the other hand, 31 cases were false negatives. The BG test was performed in the early stage of PCP in which few *P. jirovecii* cysts were destroyed, and thus, the BG level did not increase. One-third of these 31 patients were taking a preventive dose of sulfanilamide for PCP; sulfanilamide prevention may also reduce the level of the BG test.

Studies on the accuracy of LDH in the diagnosis of PCP are rare^6,13,17,20^. Deng C performed a systematic review and concluded that the sensitivity of LDH in the diagnosis of PCP is above 80%, and the value is especially high in the AIDS group, but the specificities varied greatly, from 6 to 85%^17^. Our study was the first aimed at non-HIV immunocompromised patients, and the sensitivity and specificity were meaningful. There were still 31 false-negative cases and 23 false-positive cases. We reviewed the false-negative cases and found that LDH was tested in the early stage of the disease when patients had a low *P. jirovecii* burden. The majority of the LDH values were close to the cut-off value (20/31 of the LDH levels were over 200 U/L, which is close to the cut-off value of 296 U/L). In cases of false positives, although we excluded cases with abnormal liver function and haematological malignancies, LDH is widely expressed in human tissues and is released when the cytoplasmic cell membrane is damaged. The LDH result could be influenced by other lung infections and various extrapulmonary disorders.

To our knowledge, this was the largest study of a novel diagnostic test for PCP among non-HIV-infected individuals. Since popularizing BG and LDH testing, we have found that the yield of PCP diagnoses has increased, while empirically treated cases and the need for bronchoscopy have been reduced. We hope that our study results can help BG and LDH become helpful adjunctive tests for PCP diagnosis in non-HIV immunocompromised patients.

At the same time, the study has some limitations. First, the sensitivity and specificity of BG and LDH to distinguish proven PCP from probable PCP are poor. Second, we did not analyse the relationship between the serum marker level and the *P. jirovecii* burden, so the response to therapy based on BG or LDH should be evaluated. Third, cases were excluded based on the criteria given above, but this method may overemphasize the specificity of the tests; unfortunately, the sample sizes of patients who both matched the exclusion criteria and took the BG or LDH test were small, and we did not analyse the influence of these factors. Finally, because of the different inclusion and exclusion criteria of LDH and BG group, the target populations are also different. The sample size of population meeting the inclusion and exclusion criteria of these two groups was too small to analyse, there is no combined analysis for both BG and LDH in this study. Furtherly we could collect enough cases to analyse the BG level, LDH level and even more related diagnostic tests comprehensively, in this way, the PCP might be recognized more specifically and sensitively in the immunocompromised patients.

In summary, we found that in HIV-uninfected immunocompromised patients with pneumonia symptoms, positive BG and LDH tests had a high predictive value for the diagnosis of PCP. We suggest that serum BG and LDH levels can provide helpful diagnostic support for PCP in this population, especially in settings where invasive testing for PCP is either unavailable or expensive.

## Supplementary Information

Supplementary Information.

## Data Availability

The datasets used and analysed during the current study are available from the corresponding author on reasonable request.

## Code availability

11578.
