# Supplementary material for: Diagnostic accuracy of the 1,3-beta-d-glucan test and lactate dehydrogenase for pneumocystis pneumonia in non-HIV patients
Source: Sci Rep. 2021 Apr 29;11:9226. doi: 10.1038/s41598-021-88729-z (PMC8085008; doi:10.1038/s41598-021-88729-z)
Supplement: Supplementary file 1 — Supplementary Information. [file 41598_2021_88729_MOESM1_ESM.docx]

**Diagnostic accuracy of the 1,3-beta-D-glucan test and lactate dehydrogenase for pneumocystis pneumonia in non-HIV patients**

Ruixue Sun, Dan Lv, Meng Xiao, Li Zhang, Jun Xu, Xuezhong Yu, Huadong Zhu, Jing Yang

Ruixue Sun: Emergency Department, State Key Laboratory of Complex Severe and Rare Diseases, Peking Union Medical College Hospital, Chinese Academy of Medical Science and Peking Union Medical College, Beijing, China.; srxsunruixue@163.com

Dan Lv: Beijing Emergency Medical Center, Beijing, China; 756440708@qq.com

Meng Xiao: Laboratory Department, State Key Laboratory of Complex Severe and Rare Diseases, Peking Union Medical College Hospital, Chinese Academy of Medical Science and Peking Union Medical College, Beijing, China; cjtcxiaomeng@aliyun.com

Li Zhang: Laboratory Department, State Key Laboratory of Complex Severe and Rare Diseases, Peking Union Medical College Hospital, Chinese Academy of Medical Science and Peking Union Medical College, Beijing, China; zhanglipumchlab@163.com

Jun Xu: Emergency Department, State Key Laboratory of Complex Severe and Rare Diseases, Peking Union Medical College Hospital, Chinese Academy of Medical Science and Peking Union Medical College, Beijing, China; pumchxujun@126.com

Xuezhong Yu: Emergency Department, State Key Laboratory of Complex Severe and Rare Diseases, Peking Union Medical College Hospital, Chinese Academy of Medical Science and Peking Union Medical College, Beijing, China; srxgongzuoriji@163.com

Huadong Zhu: Emergency Department, State Key Laboratory of Complex Severe and Rare Diseases, Peking Union Medical College Hospital, Chinese Academy of Medical Science and Peking Union Medical College, Beijing, China; Zhuhuadong1970@126.com

*Corresponding Author:

*Jing Yang: Emergency Department, State Key Laboratory of Complex Severe and Rare Diseases, Peking Union Medical College Hospital, Chinese Academy of Medical Science and Peking Union Medical College, Beijing, China; pumchyangjing@126.com

Supplemental Table 1

The mean level of BG in NO PCP, probable PCP and proven PCP groups

|  | mean | SD | median | min | max |
| --- | --- | --- | --- | --- | --- |
| NO PCP | 65.03 | 105.32 | 27.95 | 9.00 | 580.00 |
| Probable | 983.22 | 1108.76 | 580.00 | 14.00 | 6883.00 |
| Proven | 1220.09 | 832.01 | 1106.95 | 63.00 | 3190.00 |

Supplemental Table 2

The mean level of LDH in NO PCP, probable PCP and proven PCP groups

|  | mean | SD | median | min | max |
| --- | --- | --- | --- | --- | --- |
| NO PCP | 297.62 | 126.40 | 263.00 | 44.00 | 622.00 |
| Probable | 547.88 | 280.81 | 494.00 | 132.00 | 2098.00 |
| Proven | 854.24 | 1676.37 | 512.00 | 202.00 | 10024.00 |

Supplemental Table 3

Differences of BG levels in NO PCP, probable PCP and proven PCP groups

| classification |  | SE | P | 95% CI |
| --- | --- | --- | --- | --- |
| NO PCP | probable | 155.20 | 0.000 | -1224.00~-612.37 |
|  | proven | 236.96 | 0.000 | -1622.00~-688.12 |
| probable | NO PCP | 155.20 | 0.000 | 612.37~1224.00 |
|  | proven | 209.32 | 0.259 | -649.34~175.59 |
| proven | NO PCP | 236.96 | 0.000 | 688.12~1622.00 |
|  | probable | 209.32 | 0.259 | -175.59~649.34 |

(SE: Standard error, CI: confidence interval)

Supplemental Table 4

Differences of LDH levels in NO PCP, probable PCP and proven PCP groups

| classification |  | SE | P | 95% CI |
| --- | --- | --- | --- | --- |
| NO PCP | probable | 102.5807 | 0.015 | -452.309~-48.212 |
|  | proven | 143.8549 | 0.000 | -839.964~-273.275 |
| probable | NO PCP | 102.5807 | 0.015 | 48.212~452.309 |
|  | proven | 123.8362 | 0.014 | -550.274~-62.444 |
| proven | NO PCP | 143.8549 | 0.000 | 273.275~839.964 |
|  | probable | 123.8362 | 0.014 | 62.444~550.274 |

Supplemental Table 5

BG performance at distinguishing NO PCP, probable PCP and proven PCP

|  | prob vs. prov | prob vs. no PCP | prov vs. no PCP | prob/prov vs. no PCP |
| --- | --- | --- | --- | --- |
| cutoff | 598.5 | 110 | 285.8 | 144.1 |
| true positive | 18 | 44 | 22 | 45 |
| false negative | 76 | 22 | 2 | 25 |
| true negative | 79 | 133 | 48 | 154 |
| false positive | 6 | 6 | 2 | 5 |
| sensitivity% (95% CI) | 75（53-89） | 88（75-95） | 92（72-99） | 90 (77-96) |
| specificity% (95% CI) | 51（43-59） | 86（79-91） | 96（85-99） | 86 (80-91) |
| % agreement | 54 | 86 | 95 | 87 |
| kappa | 0.12 | 0.67 | 0.88 | 0.67 |
| positive LR | 1.53（1.15-2.23） | 6.20（4.15-9.25） | 22.9（5.9-89.6） | 6.44 (4.43-9.38) |
| negative LR | 0.49（0.24-0.99） | 0.14（0.07-0.30） | 0.09（0.02-0.33） | 0.12 (0.05-0.27) |

Supplemental Table 6

LDH performance at distinguishing NO PCP, probable PCP and proven PCP

|  | prob vs. prov | prob vs. no PCP | prov vs. no PCP | prob/prov vs. no PCP |
| --- | --- | --- | --- | --- |
| cutoff | 482 | 296 | 379 | 363 |
| true positive | 22 | 35 | 28 | 40 |
| false negative | 84 | 19 | 12 | 43 |
| true negative | 79 | 144 | 41 | 136 |
| false positive | 11 | 18 | 5 | 10 |
| sensitivity% (95% CI) | 67（48-81） | 66（52-78） | 85（67-94） | 80 (66-89) |
| specificity% (95% CI) | 48（41-56） | 88（82-93） | 77（63-87） | 76 (69-82) |
| % agreement | 52 | 83 | 80 | 77 |
| kappa | 0.08 | 0.54 | 0.60 | 0.45 |
| positive LR | 1.30（0.97-1.72） | 5.67（3.56-9.02） | 3.75（2.23-6.29） | 3.33 (2.48-4.47) |
| negative LR | 0.69（0.42-1.13） | 0.38（0.26-0.56） | 0.20（0.09-0.44） | 0.26 (0.15-0.46) |

The primer and probe were PAZ-F (5’- CTTAAAATAAATAATCAGACTATGTGCGATAAG-3’), PAZ-R(5’-GGAGCTTTAATTACTGTTCTGGGC-3’), PAZ-P (5’-FAM-TAGATAGTCGAAAGGGAAA-MGB-3’) synthesized by Sangon Biotech.
